# Emptying and filling a tunnel bronze†
†Electronic supplementary information (ESI) available. See DOI: 10.1039/c4sc03748k



**DOI:** 10.1039/c4sc03748k

**Published:** 2015-01-13

**Authors:** Peter M. Marley, Tesfaye A. Abtew, Katie E. Farley, Gregory A. Horrocks, Robert V. Dennis, Peihong Zhang, Sarbajit Banerjee

**Affiliations:** a Department of Chemistry , Texas A&M University , College Station , TX 77842-3012 , USA . Email: banerjee@chem.tamu.edu; b Department of Physics , University at Buffalo , The State University of New York , Buffalo , New York 14260 , USA

## Abstract

The classical orthorhombic layered phase of V_2_O_5_ has long been regarded as the thermodynamic sink for binary vanadium oxides and has found great practical utility as a result of its open framework and easily accessible redox states. Herein, we exploit a cation-exchange mechanism to synthesize a new stable tunnel-structured polymorph of V_2_O_5_ (ζ-V_2_O_5_) and demonstrate the subsequent ability of this framework to accommodate Li and Mg ions. The facile extraction and insertion of cations and stabilization of the novel tunnel framework is facilitated by the nanometer-sized dimensions of the materials, which leads to accommodation of strain without amorphization. The topotactic approach demonstrated here indicates not just novel intercalation chemistry accessible at nanoscale dimensions but also suggests a facile synthetic route to ternary vanadium oxide bronzes (M_*x*_V_2_O_5_) exhibiting intriguing physical properties that range from electronic phase transitions to charge ordering and superconductivity.

## Abstract 2

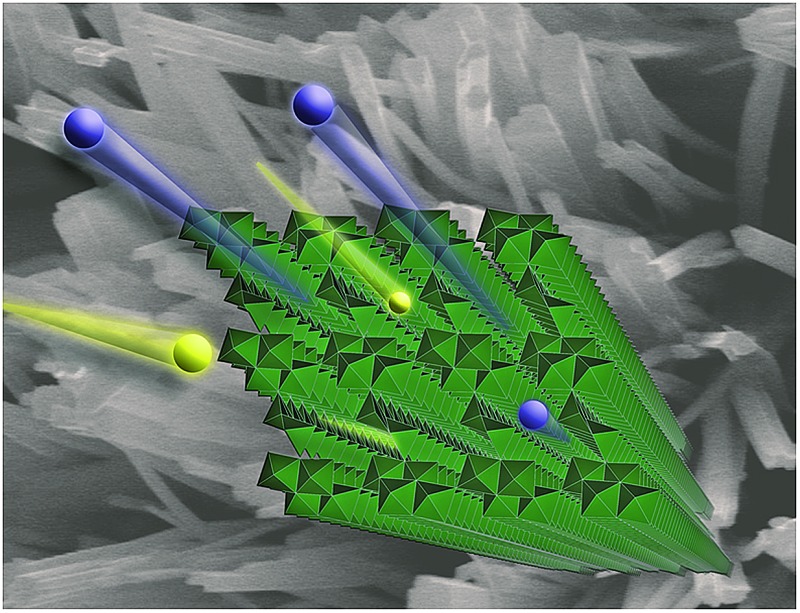
We report the synthesis of a new tunnel-structured polymorph of V_2_O_5_ (ζ-V_2_O_5_) synthesized by topotactic ion extraction.

## Introduction

**Fig. 1:**
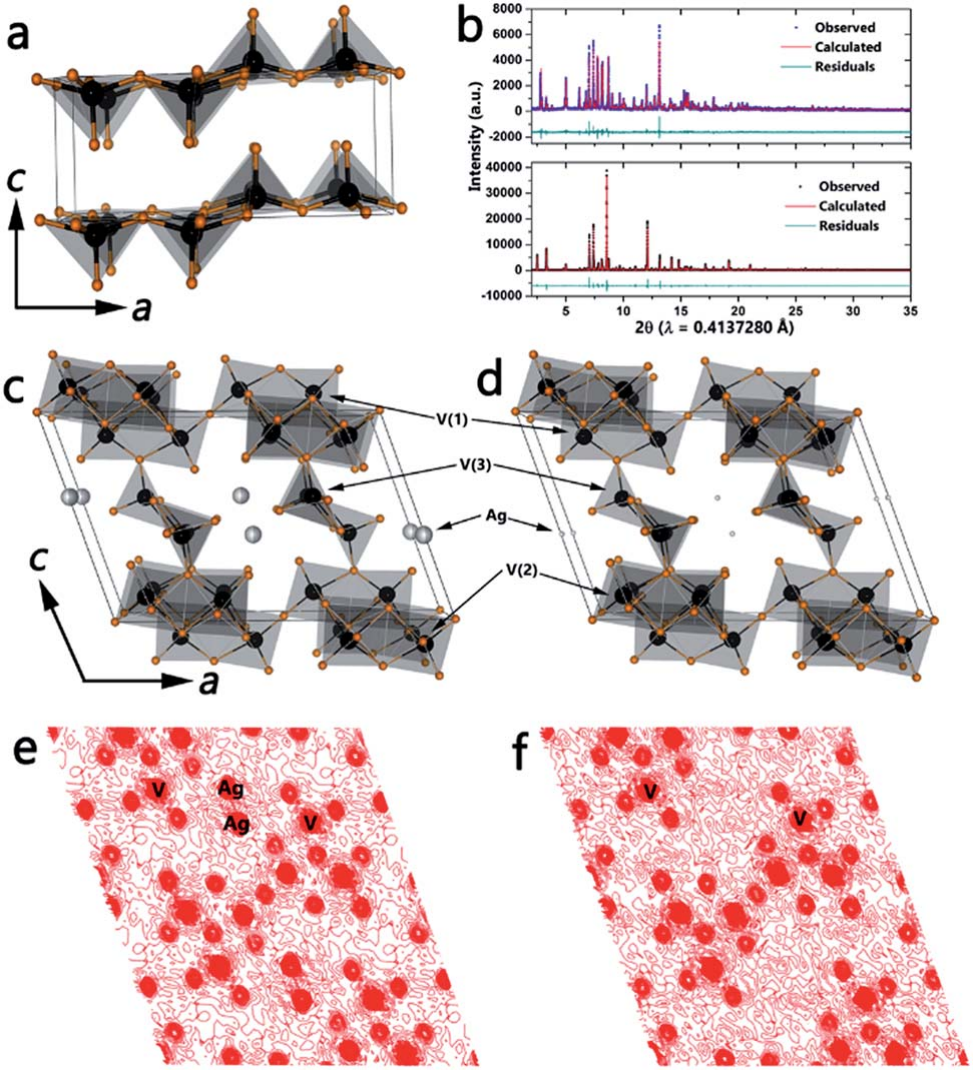
(a) Crystal structure of the orthorhombic layered structure of α-V_2_O_5_. High-resolution synchrotron powder X-ray diffraction pattern (*λ* = 0.4137280 Å) acquired for β-Ag_*x*_V_2_O_5_ (top panel, b) and leached ζ-V_2_O_5_ (bottom panel, b). Blue squares (top) and black circles (bottom) show the observed reflections for β-Ag_*x*_V_2_O_5_ and ζ-V_2_O_5_, respectively. The red and teal lines are the calculated diffraction patterns and residuals, respectively. (c) The refined β-Ag_*x*_V_2_O_5_ structure indicating the three vanadium atoms (black) bonded to oxygen (orange) and Ag-ions (grey) residing within tunnel sites. (d) The crystal structure of Ag-leached ζ-V_2_O_5_ refined from the diffraction pattern in (b). Upon reaction with HCl, the Ag-ions are almost entirely removed. The Fourier maps of the observed electron density projected on the (010) lattice plane for β-Ag_*x*_V_2_O_5_ and ζ-V_2_O_5_ are shown in (e) and (f). The electron density from the Ag-ions is clearly almost completely eliminated in (f).

It was in 1867 that the British chemist Sir Henry Enfield Roscoe first outlined the various binary oxides of vanadium resulting from the facile accessibility of multiple oxidation states of this transition metal as part of his Bakerian lecture to the Royal Society; in doing so, he set the record straight on the formula of the end-member “vanadic acid” V_2_O_5_, which had previously erroneously been described by Berzelius to have the formula VO_3_.^1^ As it turns out, the facile stabilization of mixed valence vanadium sites and the accommodation of oxygen vacancies through crystallographic shear allows for a much richer phase diagram than he originally anticipated even for just binary vanadium oxides with the occurrence of numerous Magneli-type phases.^2^ However, it took more than a century since the initial studies by Roscoe for the first accurate elucidation of the crystal structure of V_2_O_5_, which with pentavalent vanadium is the “thermodynamic sink” in this system, and not surprisingly, is the most common naturally occurring oxide ore of vanadium (found typically in volcanic craters) and an ubiquitous industrial precursor for the preparation of ferrovanadium. Byström *et al.* first established the now classic orthorhombic layered structure of V_2_O_5_ with a space group of *Pmmn* derived from square pyramidally coordinated vanadium-centered building blocks; in this structure, the VO_5_ polyhedra are knitted together with shared edges forming a zig-zag chain along the *b*-axis and are arrayed with shared corners along the crystallographic *a* axis (Fig. 1a).^3^ Galy further established that the long V–O distance was much too long to be a bonding interaction and thus proposed that the 2D infinite V_2_O_5_ sheets were held together in the *c* direction by relatively weak van der Waals' interactions.^2c,4^ The combination of an open layered framework that is only weakly interacting in the *c* direction and the facile accessibility of multiple oxidation states underpins the use of V_2_O_5_ for a broad range of applications ranging from cathodes for Li-ion batteries, and catalysts for selective oxidation to electrochromic elements, actuators, anti-fouling films, and photodectors.^5,6^

Despite the technological interest derived from this remarkable combination of an open framework, facile redox characteristics, and tolerance to defects, it appears that orthorhombic V_2_O_5_ is by far the most thermodynamically accessible phase under ambient conditions. There is only one notable metastable phase of V_2_O_5_ (space group = *Pmna*) that is derived from de-intercalation of Li-ions from a puckered (but still layered) lithiated phase, γ-Li_*x*_V_2_O_5_ (*x* > 1).^7*a*^ This puckered γ-V_2_O_5_ phase is stable up to *ca.* 340 °C where it transforms to the thermodynamically stable orthorhombic V_2_O_5_ structure.^7*a*^ A high-pressure β-phase has also been reported.^7*b*^ In this work, we report the stabilization of a novel tunnel-structured ζ-V_2_O_5_ (space group: *C*2/*m*) phase based on the hydrothermal de-intercalation of Ag^+^ ions from nanowires of a β-Ag_*x*_V_2_O_5_ tunnel structure. The tunnel framework appears to be stable to temperatures of up to 600 °C and remains available for subsequent intercalation with Li and Mg-ions.

## Synthesis and characterization

β-Ag_*x*_V_2_O_5_ nanowires were synthesized by reacting stoichiometric amounts of silver acetate (Sigma Aldrich) and V_2_O_5_ (Sigma Aldrich) with 16 mL H_2_O (*ρ* = 18 MΩ cm^–1^) in a Teflon-lined acid digestion vessel at 210 °C for 72 h. The resulting solid was washed with water and allowed to dry in air. The leached ζ-V_2_O_5_ structure was synthesized by hydrothermally treating the β-Ag_*x*_V_2_O_5_ nanowires with 15 mL of 0.71 M HCl at 210 °C for 24 h. After allowing the reaction to cool the solid was filtered and washed with copious amounts of water and isopropanol and then allowed to dry in air overnight. Reinsertion of Li-ions into the empty ζ-V_2_O_5_ framework was performed by mixing stoichiometric amounts of leached ζ-V_2_O_5_ nanowires and *n*-butyllithium for 96 h in toluene under an Ar atmosphere. The resulting solid was filtered, washed with copious amounts of water, and allowed to dry in air overnight. β-Mg_*x*_V_2_O_5_ nanowires were synthesized by stirring Mg nanoplatelets (dimensions of 100–500 nm in diameter, prepared by the reduction of CH_3_MgCl by lithium naphthalide as reported in our previous work)^8^ with the leached ζ-V_2_O_5_ nanowires in 20 mL of H_2_O at room temperature for 48 h. The resulting solid was washed with copious amounts of water and allowed to dry in air.

Synchrotron powder X-ray diffraction data were acquired in transmission geometry at beamline 11-BM of the Advanced Photon Source at Argonne National Laboratory. Rietveld refinements were performed using the GSAS/EXPGUI software.^9^ *In situ* heating of the leached ζ-V_2_O_5_ phase was performed using a Rigaku Ultima IV diffractometer with Cu Kα radiation and an Ultima IV HT 1500 temperature attachment with a PTC-30 programmable temperature controller and a platinum sample holder from room temperature to 600 °C under an ambient atmosphere. A 10 °C min^–1^ ramp rate was used with a hold time of 30 min before acquiring each pattern. The nanowires were further examined by scanning electron microscopy (SEM, Hitachi SU-70, 20 kV, equipped with an energy dispersive X-ray detector for elemental composition) and transmission electron microscopy (TEM, JEOL 2010, 200 kV). Chemical analysis was performed by inductively coupled plasma mass spectrometry (ICP-MS) after digesting the samples in 10% aqueous solutions of nitric acid. X-ray photoelectron spectroscopy was performed on a Phi 5000 VersaProbe instrument with monochromatic Al Kα X-rays and with charge neutralization of the samples. Near-edge X-ray absorption fine structure (NEXAFS) measurements were carried out at the National Institute of Standards and Technology beamline U7A of the National Synchrotron Light Source of Brookhaven National Laboratory with a toroidal mirror spherical grating monochromator using a 1200 lines per mm grating and an energy resolution of 0.1 eV. NEXAFS data were collected in partial electron yield (PEY) mode with a channeltron multiplier near the sample surface using the detector at –200 V bias to enhance surface sensitivity. The PEY signal was normalized by the drain current of a clean gold mesh located along the path of the incident X-rays. All data was collected with a standard reference of metallic vanadium foil for energy calibration. DFT calculations were performed using the QUANTUM ESPRESSO package using the generalized gradient approximation with Perdew–Burke–Ernzerhof functionals.^10^ Ultrasoft pseudopotentials were used to describe the electron–ion interactions.^11,12^

## Results and discussion

Ion-exchange and other topotactic reactions are commonly used to access a variety of compounds that cannot directly be synthesized from their constituent elements owing to insurmountable thermodynamic considerations; examples of the synthetic utility of these methods span the range from novel perovskites to semiconductor quantum dots and chalcogenides.^13,14^ Scaling materials to nanoscale dimensions further provides access to crystal structures that may not be readily stabilized at room temperature in bulk form. While some of these structures are metastable, others are in fact thermodynamically stable either due to surface energy considerations or because strain effects can be better accommodated at nanoscale dimensions.^15^ Indeed, the ζ-V_2_O_5_ phase isolated here appears to be an unusual thermodynamically stable polymorph.

The novel ζ-V_2_O_5_ structure has been derived from the almost complete leaching of Ag-ions from a ternary vanadium oxide bronze β-Ag_0.33_V_2_O_5_ as per:

1 β-Ag_0.33_V_2_O_5_ (s) + 0.29HCl (aq.) + 0.145O_2_ → ζ-(Ag_0.04_)V_2_O_5_ (s) + 0.29AgCl (s) + 0.145H_2_O

The ternary vanadium oxide bronzes of which β-Ag_0.33_V_2_O_5_ is an example are important in their own right and exhibit remarkable first- and second-order electronic phase transitions such as colossal metal–insulator transitions (induced as a function of temperature, voltage, or cation concentration), superconductivity, and charge and spin density waves.^16,17^ Cation intercalation and concomitant partial reduction of the V_2_O_5_ frameworks yields charge ordered networks that extend along the length of the vanadium oxide chains, thereby affording excellent model systems for examination of strong electron correlation.^18^ The size, polarizability, extent of covalency of cation–oxygen interactions, and cation concentration dictates the structure adopted by the ternary and quaternary vanadium oxides with single-layered, highly puckered layered, double-layered, and tunnel frameworks being some of the more commonly adopted frameworks.^2a,c,16c,19,20^ Indeed, the ability to fill empty tunnels of the novel ζ-V_2_O_5_ phase with other cations as noted below provides a versatile synthetic route for obtaining intercalated ternary vanadium oxide bronzes and for systematically tuning electron correlation.

β-Phase vanadium oxide bronzes have been extensively studied since Wadsley discovered that β-Na_*x*_V_2_O_5_ has the ability to accommodate a range of sodium concentrations.^19*a*^ Nanowires of β-Ag_0.33_V_2_O_5_ have been prepared by the hydrothermal reaction between silver acetate and V_2_O_5_ as described in the Methods section. The top panel in Fig. 1b shows the refined powder XRD pattern and Fig. 1c illustrates the refined structure (space group: *C*2/*m*), which is based on three distinct vanadium-centered polyhedra: edge-sharing V(1)O_6_ distorted octahedra, corner-sharing V(2)O_6_ distorted octahedra, and V(3)O_5_ square pyramids (see Table S1, ESI† for refined unit cell parameters and atom positions). The polyhedra form infinite chains parallel to the crystallographic *b*-axis and enclose tunnel sites wherein the intercalated Ag^+^ ions reside. A Ag stoichiometry of *x* = 0.33 (occupancy of 0.4956) yields the best fit to the powder pattern and has been further verified by energy dispersive X-ray analysis as well as XPS (Fig. S2†). Furthermore, ICP-MS analysis of β-Ag_*x*_V_2_O_5_ yields the relative concentrations of vanadium and silver to be 3.072 upon acid digestion, which results in an *x* value of 0.31 further corroborating the stoichiometry from the refinement.

The open tunnel framework of the β-phase permits removal of cations from interstitial sites while keeping the tunnel structure intact; indeed, for the nanowires this occurs without amorphization. Furthermore, the ability of the vanadium atom to occupy various coordination environments and oxidation states results in formation of the ζ-V_2_O_5_ open tunnel framework without reversion to α-V_2_O_5_.^2c,19a^ The bottom panel of Fig. 1b plots the refined powder XRD pattern of the leached phase and Fig. 1d shows the structure of the novel ζ-V_2_O_5_ phase. The open tunnel framework comprising V(1)O_6_, V(2)O_6_, and V(3)O_5_ polyhedra is still clearly retained with an almost complete removal of the interstitial Ag-ions (see Table S2, ESI† for refined unit cell parameters and atom positions). A comparison of the local vanadium–oxygen coordination environment for the three distinct vanadium atoms is shown in Table S3.† The significant elimination of the intercalated Ag^+^ cations is verified by the Fourier maps of the charge density along the (010) planes depicted in Fig. 1e and f. The *x* value determined from the refinement (and corroborated *via* energy-dispersive X-ray analysis and XPS) is much lower than was previously thought to be necessary to stabilize the β-phase framework.^2c,19a^ ICP-MS analysis of the ζ-V_2_O_5_ structure gives relative concentrations of vanadium and silver to be 16.08; this confirms the stoichiometry from the refinement and the removal of the interstitial Ag-ions (*x* = 0.06). Fig. S1 (ESI†) shows the marked change in color accompanying the leaching of Ag ions from β-Ag_0.33_V_2_O_5_, suggesting recovery of the orange color characteristic of V^5+^ cations. Fig. S2 (ESI†) shows V 2p X-ray photoelectron spectroscopy data acquired for β-Ag_0.33_V_2_O_5_ and the leached ζ-V_2_O_5_ phase. Upon removal of Ag-ions, it is expected that the number of electrons localized on the vanadium oxide framework will decrease as verified by the pronounced decrease in the intensity of the V^4+^ shoulder. The inclusion of some protons in the tunnels cannot be entirely ruled out but the recovery of the orange color and the predominant contributions from V^5+^ in both XPS and NEXAFS spectra are suggestive of oxidation of V^4+^ sites within the vanadium oxide bronze back to V^5+^.

**Fig. 2:**
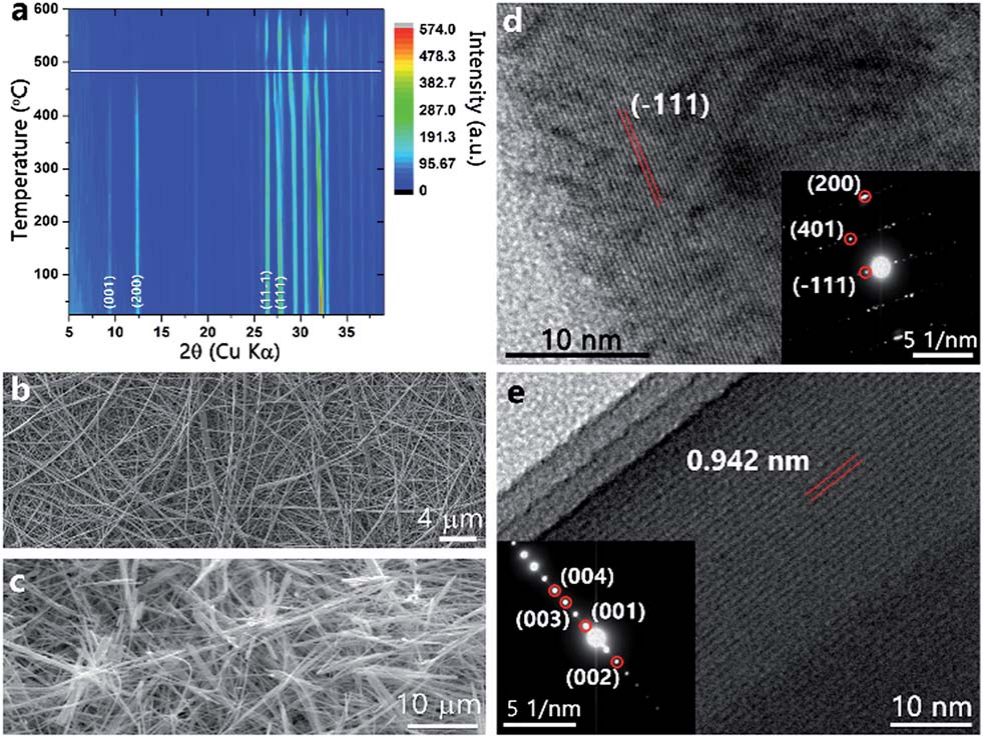
(a) Contour plot of XRD reflections of ζ-V_2_O_5_ measured as a function of temperature from room temperature (*ca.* 20 °C) up to 600 °C. Reflections characteristic of orthorhombic V_2_O_5_ are not observed in this temperature range. (b) SEM image of β-Ag_*x*_V_2_O_5_ illustrating the nanowire morphology. (c) SEM image of the nanowires after leaching of Ag ions. (d) Lattice-resolved high-resolution TEM image of a β-Ag_*x*_V_2_O_5_ nanowire with a lattice spacing corresponding to the separation between the (–111) planes of the refined structure. The inset shows the corresponding indexed SAED pattern. (e) High-resolution TEM image of a single ζ-V_2_O_5_ nanowire with a lattice spacing of 0.942 nm which matches the spacing between (001) planes of the leached ζ-V_2_O_5_ structure. The inset shows the indexed SAED pattern of the same nanowire.

The thermal stability of ζ-V_2_O_5_ has been studied by temperature-dependent powder X-ray diffraction. Fig. 2a shows a contour plot of the reflections as a function of temperature from 20 to 600 °C under an ambient atmosphere. ζ-V_2_O_5_ is clearly stable up to at least 490 °C (demarcated by a white line for clarity) at which point the low 2*θ* reflections (001) and (200) decrease in intensity indicating loss of long-range order. The reflections suggest that the leached structure is not merely a metastable phase of V_2_O_5_. Differential scanning calorimetry (DSC) is a sensitive probe of dehydration of intercalated water molecules in hydrated vanadium oxides; in past work, we have observed a pronounced endothermic feature at *ca.* 250 °C corresponding to the removal of interstitial water molecules.^18*c*^ However, DSC measurements performed on the leached ζ-V_2_O_5_ structure show no calorimetric signatures corresponding to removal of water molecules from the interstitial tunnel sites.

Fig. 2b shows a SEM image of the starting β-Ag_*x*_V_2_O_5_ nanowires, which have lengths up to and greater than 10 μm and lateral dimensions of 150 ± 9 nm. The nanowire morphology is retained for the leached ζ-V_2_O_5_ phase after removal of Ag-ions from the interstitial tunnel sites as indicated in Fig. 2c without any discernible fragmentation. The HRTEM image in Fig. 2d along with the corresponding selected area electron diffraction (SAED) pattern indicates that the initial β-Ag_*x*_V_2_O_5_ nanowires are single crystalline. Fig. 2e and the SAED pattern in the inset further indicate that the leached ζ-V_2_O_5_ remain single crystalline upon removal of Ag-ions. The lattice spacing of 0.942 nm closely matches the spacing between the (001) lattice planes of the refined structure (Fig. 1d). The electron microscopy observations suggest that the shorter diffusion path lengths available at the nanoscale (as compared to the bulk) facilitate rapid de-intercalation of the Ag^+^ ions, allowing for stabilization of the empty tunnel framework. The ability of the nanowires to accommodate strain also likely plays a significant role in permitting the removal of silver cations without destruction of the morphology or amorphization. Intriguingly, the role of finite size here is some-what different than in the case of de-intercalation of the Li_*x*_V_2_O_5_. Indeed, removal of Li-ions from nanoscale γ-Li_*x*_V_2_O_5_ results in transformation back to α-V_2_O_5_ unlike in the bulk wherein the meta-stable γ-V_2_O_5_ structure is stabilized.^21^ From a structural perspective, the ability of the framework to remain intact upon removal of the majority of the Ag^+^ ions is likely mediated by the VO_5_ square pyramid chains that act in the same fashion as the PO_4_ tetrahedra that hold the iron oxide octahedra together upon delithiation of LiFePO_4_.^22^ Such structural stability of the tunnel framework is imperative to realize the potential of topotactic reactions and precisely manipulate the physical properties of vanadium oxide bronzes. In contrast, a control de-intercalation reaction attempted for micron-sized particles of β-Ag_0.33_V_2_O_5_ (prepared by the solid-state reaction of Ag_2_O, V_2_O_5_, and V_2_O_3_ at 650 °C) shows no evidence for deintercalation of Ag-ions from the interstitial tunnel sites.

**Fig. 3:**
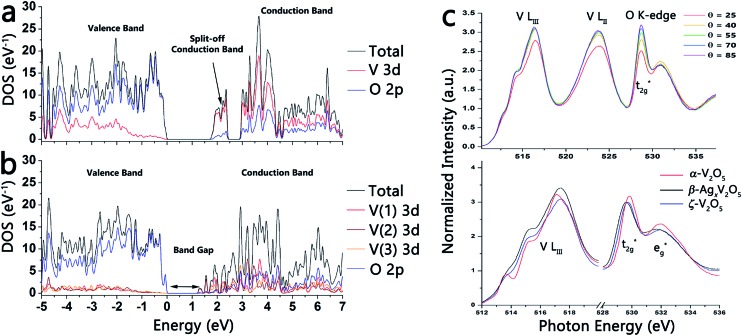
(a) Calculated density of states for α-V_2_O_5_. (b) The density of states for the ζ-V_2_O_5_ structure (the legend on the right illustrates atom-projected density of states derived from the different atoms). The DOS have been normalized per V_2_O_5_ formula unit. (c) Top panel: polarized NEXAFS measurements performed on a pressed pellet of ζ-V_2_O_5_ nanowires indicating V L_III_, V L_II_, and O K-edge resonances. Although the intensity of the t_2g_* feature is seen to increase with increasing incident photon energy, the strong anisotropy characteristic of α and δ-phase V_2_O_5_ and ternary vanadium oxides is not observed for the ζ-framework. Bottom panel: NEXAFS spectra acquired for α-V_2_O_5_ (red), β-Ag_*x*_V_2_O_5_ (black), and ζ-V_2_O_5_ (blue).

Fig. 3a and b contrasts the calculated density of states of layered orthorhombic α-V_2_O_5_ (Fig. 1a) and the novel tunnel-structured leached ζ-V_2_O_5_ phase (Fig. 1d). For both polymorphs, the valence band is mostly O 2p in character, whereas the conduction band is primarily V 3d in character.^23^ Eyert and co-workers have calculated a bandgap of *ca.* 1.7 eV for orthorhombic α-V_2_O_5_; a notable consequence of the anisotropic layered structure of α-V_2_O_5_ is the appearance of a split off conduction band about 0.35 eV below the primary conduction band that is essentially derived from V 3d_*xy*_ orbitals.^23*b*^ The V 3d_*xy*_ origin of the split-off conduction bands has been verified in previous polarized near-edge X-ray absorption fine structure (NEXAFS) spectroscopy studies of α-V_2_O_5_ nanowire arrays.^23*a*^ In contrast, the bandgap calculated using the refined coordinates for the ζ-V_2_O_5_ structure (Fig. 3b) is *ca.* 1.1 eV. Owing to the more complex geometric structure of this novel tunnel phase, no distinct split-off conduction band feature is predicted. Fig. S3 (ESI†) shows the atom-projected density of states calculated for the three distinct V(1), V(2), and V(3) atoms of ζ-V_2_O_5_; the contributions from each of the five V 3d orbitals has been deconvoluted and it is apparent that the lowest energy conduction band states in ζ-V_2_O_5_ includes contributions from V(1) 3d_*xy*_, V(1) 3d_*zy*_, V(2) 3d_*xy*_, V(2) 3d_*zy*_, and V(3). The complex tunnel structure (compared to the more simple orthorhombic layered structure of α-V_2_O_5_) thus gives rise to a very different bonding motif and electronic structure.^24^ More recent DFT calculations of α-V_2_O_5_ from Da Silva and co-workers in the LDA and GGA approximations suggest bandgaps of 2.21 and 2.27 eV for α-V_2_O_5_ that are closer to the optically measured values.^25^ The novel tunnel-structured phase clearly has a much smaller bandgap. The top panel in Fig. 3c depicts polarized NEXAFS spectra acquired for a pressed pellet of ζ-V_2_O_5_ nanowires and illustrates the reduced anisotropy of the lowest energy conduction band states for both the V L_III_-edge region and the O K-edge regions; in contrast, orthorhombic V_2_O_5_ and other layered δ-M_*x*_V_2_O_5_ phases show a pronounced modulation of t_2g_ : e_g_* intensity ratios at the O K-edge and clear delineation of a split-off band at the V L_III_-edge with varying polarization.^2a,18b^ The bottom panel in Fig. 3c contrasts the NEXAFS spectra of α-V_2_O_5_ (red) with β-Ag_*x*_V_2_O_5_ (black) and ζ-V_2_O_5_ (blue).

The ability of the novel ζ-V_2_O_5_ phase to serve as a synthon for preparation of ternary vanadium bronzes and for systematic modulation of strong electron correlation therein, as well as its ability to facilitate electrochemical energy storage, is predicated on the further intercalation of ions within the open framework. Here, we demonstrate that the tunnels of the ζ-V_2_O_5_ phase can be topotactically packed with Li and Mg ions as per:

2 ζ-V_2_O_5_ (s) + 0.66C_4_H_9_Li (toluene) → β′-Li_0.66_V_2_O_5_ (s) + 0.33C_8_H_18_ (butane and butane products have also been reported)

3 1.33Mg (s) + ζ-V_2_O_5_ (s) + H_2_O (l) → β-Mg_0.33_V_2_O_5_ (s) + Mg(OH)_2_ (aq.) + H_2_ (g)

**Fig. 4:**
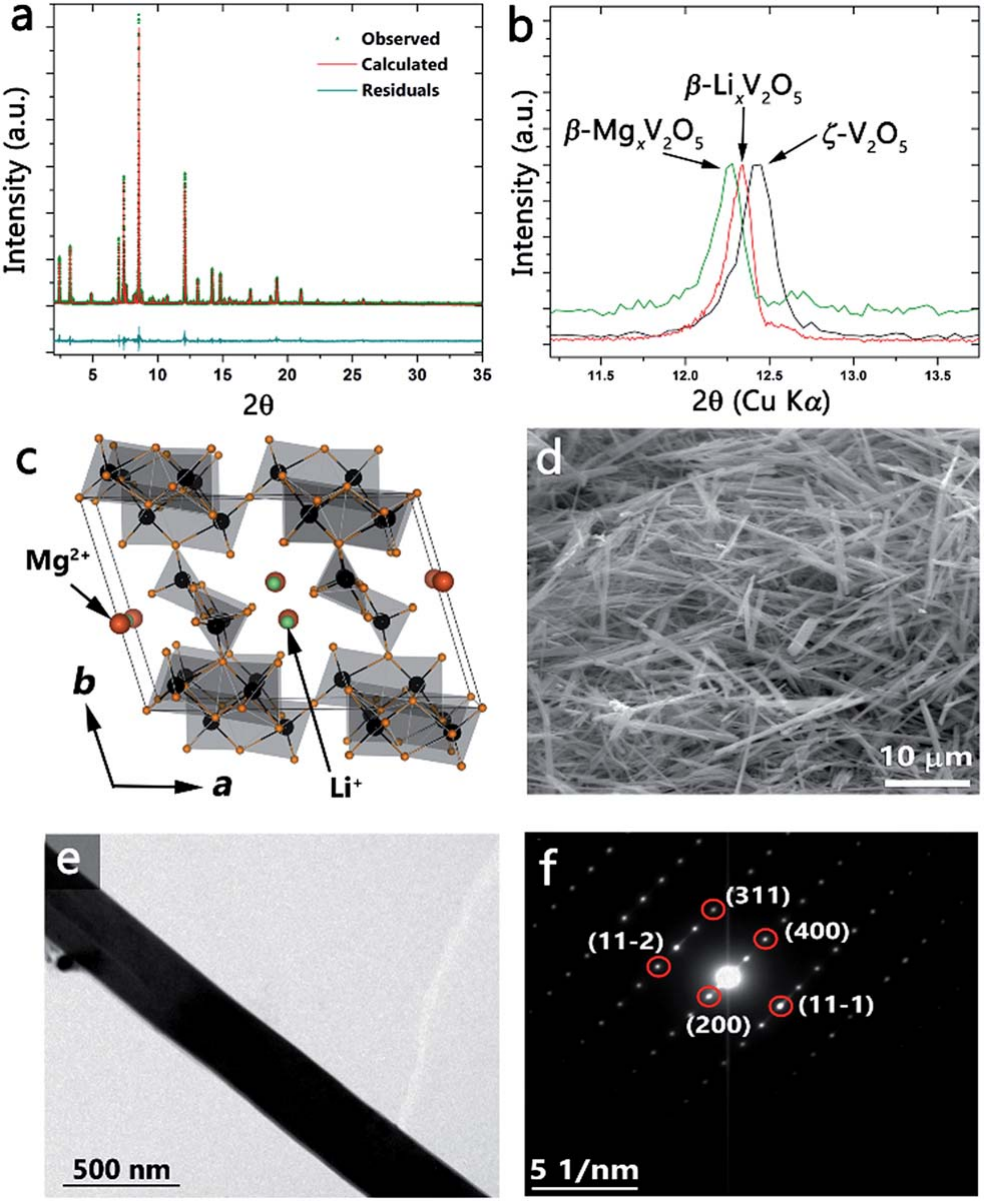
(a) Synchrotron XRD pattern obtained after reacting ζ-V_2_O_5_ with *n*-butyl-Li. The calculated pattern (red line) matches the observed reflections (green) as per the residual plot (teal line). The calculated pattern is refined with Li-ions occupying the β′-position in the tunnel framework. (b) XRD patterns obtained using Cu Kα radiation for ζ-V_2_O_5_ (black line), β′-Li_*x*_V_2_O_5_ (red line), and β-Mg_*x*_V_2_O_5_ (green line) showing the change in the (200) reflection of the β-phase. The shift in the (200) reflection to lower 2*θ* (larger *d*-spacing) is consistent with increasing ionic radii of the inserted metal cation. (c) Crystal structure of the β-phase framework depicting the different sites occupied by Li^+^ (β′-sites) and Mg^2+^ (β-sites). SEM (d) and TEM (e) images of the nanowires after insertion of Li-ions showing retention of the nanowire morphology. (f) SAED pattern for a single nanowire indexed to the β′-Li_*x*_V_2_O_5_ structure indicating the single crystalline nature of the nanowires.

Fig. 4a shows the synchrotron powder XRD pattern obtained after reaction of ζ-V_2_O_5_ with a stoichiometric amount of *n*-butyl-Li for 96 h at room temperature. The refined pattern is shown in red and the refined structure is illustrated in Fig. 4c. Table S4 (ESI†) lists the structural parameters of the β′-Li_0.66_V_2_O_5_ refined structure. Upon intercalation, the Li-ions (green) occupy the cation sites characteristic of the β′-phase owing to their small size (shifted by *b*/2 from the β-phase as illustrated in Fig. 4c). A comparison of the ζ-V_2_O_5_ pattern with that of the lithiated β′-Li_*x*_V_2_O_5_ structure is shown in Fig. S4 (ESI†) and evidences the structural transformation despite the low scattering from Li-ions. The flexibility of the ζ-V_2_O_5_ framework allows for an expansion of the tunnel structure to accommodate Li-ions (as seen in the change in the unit cell volume increasing from 522.271 to 534.581 Å^3^). The nanowire morphology is entirely retained after insertion of lithium-ions as depicted by the SEM and TEM images of Fig. 4d and e. The SAED pattern in Fig. 4f can be indexed to the refined β′-Li_*x*_V_2_O_5_ structure and illustrates the single crystalline nature is preserved after lithiation, suggesting a classical topotactic reaction.

Incorporation of Mg^2+^-ions into the open tunnel structure of ζ-V_2_O_5_ further illustrates the control over the electronic structure and properties that can be obtained through the topotactic ion-exchange pathway. Fig. 4b shows the (200) reflection for ζ-V_2_O_5_ (black), β′-Li_*x*_V_2_O_5_ (red), and β-Mg_*x*_V_2_O_5_ (green); the shift to lower 2*θ* values in progressing from ζ-V_2_O_5_ to β′-Li_*x*_V_2_O_5_ to β-Mg_*x*_V_2_O_5_ is consistent with an increase in the ionic radii of the inserted metal cation and confirms the incorporation of Mg within the interstitial tunnel sites of the β-phase. The retention of the nanowire morphology and the insertion of Mg is further verified by the SEM image and corresponding energy dispersive X-ray pattern depicted in Fig. S5.† Note that a β-phase Mg bronze has not heretofore been prepared and thus the method demonstrated here suggests the utility of the ζ-V_2_O_5_ structures to serve as synthons for preparation of novel bronzes.

## Conclusion

In conclusion, a novel polymorph of V_2_O_5_ with an open tunnel-like framework has been prepared by the topotactic de-intercalation of Ag-ions from β-Ag_0.33_V_2_O_5_ nanowires. The ability of the nanostructures to accommodate strain allows for stabilization of the tunnel framework, which is characterized by a distinctive bonding motif and an electronic structure that is significantly different from orthorhombic α-V_2_O_5_. The facile re-insertion of different metal-ions into the empty tunnel framework provides a facile synthetic route to control the charge-ordering network and electronic properties of vanadium oxide bronzes with implications for Mott field-effect transistors and memristors. Stabilizing the open tunnel structure without amorphization and being able to induce intercalation of cations further suggests applications of these materials in Li-ion and “beyond Li” batteries.^2a,26^
