# Supplementary material for: Emptying and filling a tunnel bronze
Source: Chem Sci. 2015 Jan 13;6(3):1712–8. doi: 10.1039/c4sc03748k (PMC5514409; doi:10.1039/c4sc03748k)
Supplement: Supplementary file 1 [file SC-006-C4SC03748K-s001.pdf]

Electronic Supplementary Material (ESI) for Chemical Science.
This journal is © The Royal Society of Chemistry 2015

Journal Name

RSCPublishing

ARTICLE

Supporting Information: Emptying and Filling A Tunnel Bronze

Peter M. Marley,[a] Tesfaye A. Abtew,[b] Katie E. Farley,[a] Gregory A. Horrocks,[a] Robert V.Dennis,[a] Peihong Zhang[b] and Sarbajit Banerjee[a],

SUPPORTING INFORMATION.

Table S1. Atomic and unit cell parameters for β-$Ag_xV_2O_5$.

a = 15.38202(12) Å, b = 3.614121(13) Å, c = 10.06910(6) Å, β = 109.7136(5) °, Volume = 526.9599(6) Å^3<br>χ^2 = 2.240, Rw = 6.89%					
**Atom**	***x***	***y***	***z***	**Occupancy**	**Uiso**
V(1)	0.33747(7)	0.0	0.10029(12)	1.0	0.00507(31)
V(2)	0.11636(8)	0.0	0.11883(12)	1.0	0.00724(31)
V(3)	0.28796(8)	0.0	0.40950(13)	1.0	0.00829(35)
O(1)	0.0	0.0	0.0	1.0	0.0064(15)
O(2)	0.31515(26)	0.5	0.05477(35)	1.0	0.0021(14)
O(3)	0.13415(28)	0.5	0.0786(4)	1.0	0.0017(11)
O(4)	0.44028(28)	0.0	0.2175(4)	1.0	0.0083(10)
O(5)	0.26326(24)	0.0	0.2223(4)	1.0	0.0016(11)
O(6)	0.10763(28)	0.0	0.2719(5)	1.0	0.0145(12)
O(7)	0.25679(25)	0.5	0.4261(4)	1.0	0.0065(11)
O(8)	0.40013(27)	0.0	0.4765(4)	1.0	0.0057(10)
Ag(1)	0.99688(9)	0.0	0.40389(12)	0.4956(12)	0.0229(4)

Table S2. Unit cell parameters and atomic coordinates for the ζ-V_2O_5 structure.

a = 15.27498(16) Å, b = 3.603860(17) Å, c = 10.09771(7) Å, β= 110.0222(6) °, Volume = 522.271(6) Å^3 χ^2 = 5.357, Rw = 9.87%					
**Atom**	***x***	***y***	***z***	**Occupancy**	**Uiso**
V(1)	0.33958(10)	0.0	0.10514(16)	1.0	0.0077(4)
V(2)	0.11672(10)	0.0	0.11665(15)	1.0	0.0050(4)
V(3)	0.29111(11)	0.0	0.41498(16)	1.0	0.0086(4)
O(1)	0.0	0.0	0.0	1.0	0.0099(14)
O(2)	0.81363(34)	0.0	0.0562(5)	1.0	0.0050(14)
O(3)	0.6336(4)	0.0	0.0740(5)	1.0	0.0044(12)
O(4)	0.43648(33)	0.0	0.2186(5)	1.0	0.0063(13)
O(5)	0.26572(32)	0.0	0.2229(5)	1.0	0.0018(14)
O(6)	0.1138(4)	0.0	0.2743(5)	1.0	0.0187(16)
O(7)	0.75809(34)	0.0	0.4266(5)	1.0	0.0026(13)
O(8)	0.3981(4)	0.0	0.4783(6)	1.0	0.0165(15)
Ag(1)	0.0023(7)	0.0	0.4101(10)	0.0600(9)	0.0029(8)

 This journal is © The Royal Society of Chemistry 2012

Table S3. Coordination environment around the vanadium atoms.

V-O Polyhedra	V-O	β-$Ag_xV_2O_5$ Distance (Å)	ζ-V_2O_5 Distance (Å)
$V(1)O_6$ Octahedra	V(1)-O(3)	1.991(4)	1.987(5)
	V(1)-O(5)	1.939(4)	1.899(5)
	V(1)-O(4)	1.623(4)	1.530(5)
	V(1)-O(2)	1.8674(9)	1.8747(14)
	V(1)-O(2)	1.8674(9)	1.8747(14)
	V(1)-O(2)	2.340(4)	2.349(5)
$V(2)O_6$ Octahedra	V(2)-O(1)	1.7861(12)	1.7686(14)
	V(2)-O(6)	1.591(4)	1.608(5)
	V(2)-O(3)	1.8919(11)	1.8908(16)
	V(2)-O(3)	1.8919(11)	1.8908(16)
	V(2)-O(5)	2.146(4)	2.158(5)
	V(2)-O(2)	2.326(4)	2.334(5)
$V(3)O_5$ Square Pydramid	V(3)-O(5)	1.793(4)	1.843(5)
	V(3)-O(8)	1.627(4)	1.538(5)
	V(3)-O(7)	1.8913(12)	1.8856(16)
	V(3)-O(7)	1.8913(12)	1.8856(16)
	V(3)-O(7)	1.996(4)	1.987(5)

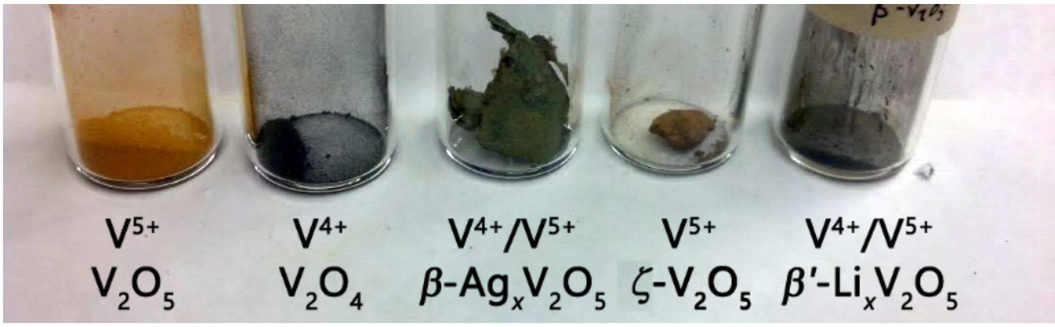

Figure S1. The leaching of Ag ions from brown—green β-$Ag_{0.33}V_2O_5$ (central vial) results in restoration of the orange—yellow coloration typical of vanadium in the +5 oxidation state (fourth from the left). Reinsertion of lithium into the tunnel structure further changes the color from orange to black as a result of partial reduction of the ζ-V_2O_5 framework to β'-$Li_xV_2O_5$.

This journal is © The Royal Society of Chemistry 2012

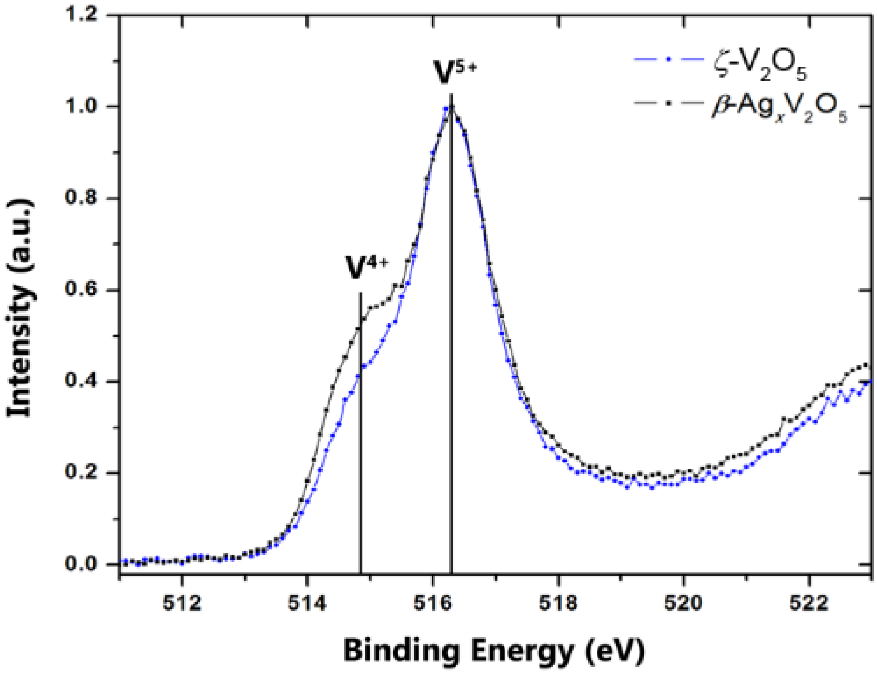

Figure S2. V $2p_{3/2}$ XPS spectra acquired for β-$Ag_xV_2O_5$ (black line) and ζ-V_2O_5 (blue line); the main feature is attributed to vanadium in a +5 oxidation state, whereas the shoulder is a result of remnant V^{4+} sites. Upon removal of silver the V^{4+} shoulder is decreased confirming the oxidation of vanadium as also visually observed in Figure S1.

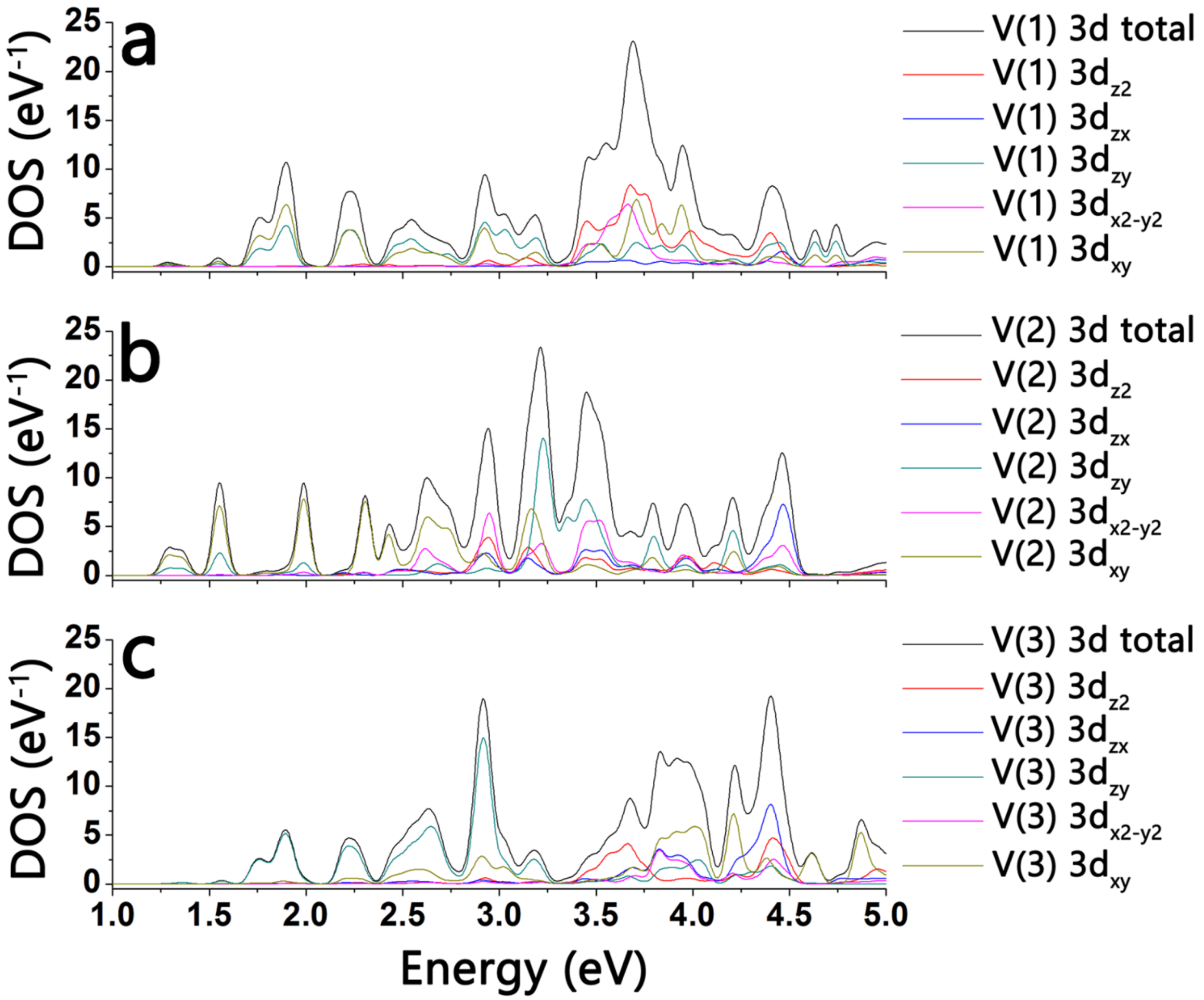

Figure S3. The onset of the conduction band, unlike α-V_2O_5, is not anisotropic and is illustrated in the orbital projected DOS for each V(1), V(2), and V(3) atom (a,b,c respectively). The lowest energy conduction band states are comprised of a number of orbitals that include: V(1) $3d_{xy}$, V(1) $3d_{zy}$, V(2) $3d_{xy}$, V(2) $3d_{zy}$, and V(3) $3d_{zy}$. The complexity of the crystal structure inherently reduces the anisotropic nature of the lowest energy conduction band states in ζ-V_2O_5.

This journal is © The Royal Society of Chemistry 2012

Table S4. Unit cell parameters and atomic positions after lithiation of ζ-V_2O_5.

a = 15.1653(4) Å, b = 3.63068(4) Å, c = 10.12429(7) Å, β = 106.4673(25) °, Volume = 534.581(20) Å^3 χ^2 = 3.067, Rw = 9.11%					
**Atom**	***x***	***y***	***z***	**Occupancy**	**Uiso**
V(1)	0.33433(25)	0.0	0.0858(4)	1.0	0.0199(13)
V(2)	0.11015(24)	0.0	0.1153(4)	1.0	0.0026(14)
V(3)	0.28539(35)	0.0	0.3932(6)	1.0	0.0416(16)
O(1)	0.0	0.0	0.0	1.0	0.0065(14)
O(2)	0.8007(8)	0.0	0.0334(13)	1.0	0.005(4)
O(3)	0.6209(7)	0.0	0.0916(11)	1.0	0.0085(25)
O(4)	0.4365(9)	0.0	0.2186(5)	1.0	0.0063(13)
O(5)	0.2770(9)	0.0	0.2276(14)	1.0	0.021(5)
O(6)	0.1138(4)	0.0	0.2743(5)	1.0	0.031(5)
O(7)	0.7440(9)	0.0	0.4312(13)	1.0	0.012(4)
O(8)	0.3943(8)	0.0	0.4630(12)	1.0	0.0145(15)
Ag(1)	0.0023	0.0	0.4101	0.0600	0.0029
Li(1)	0.0023	0.5	0.4101	1.0	0.025

This journal is © The Royal Society of Chemistry 2012

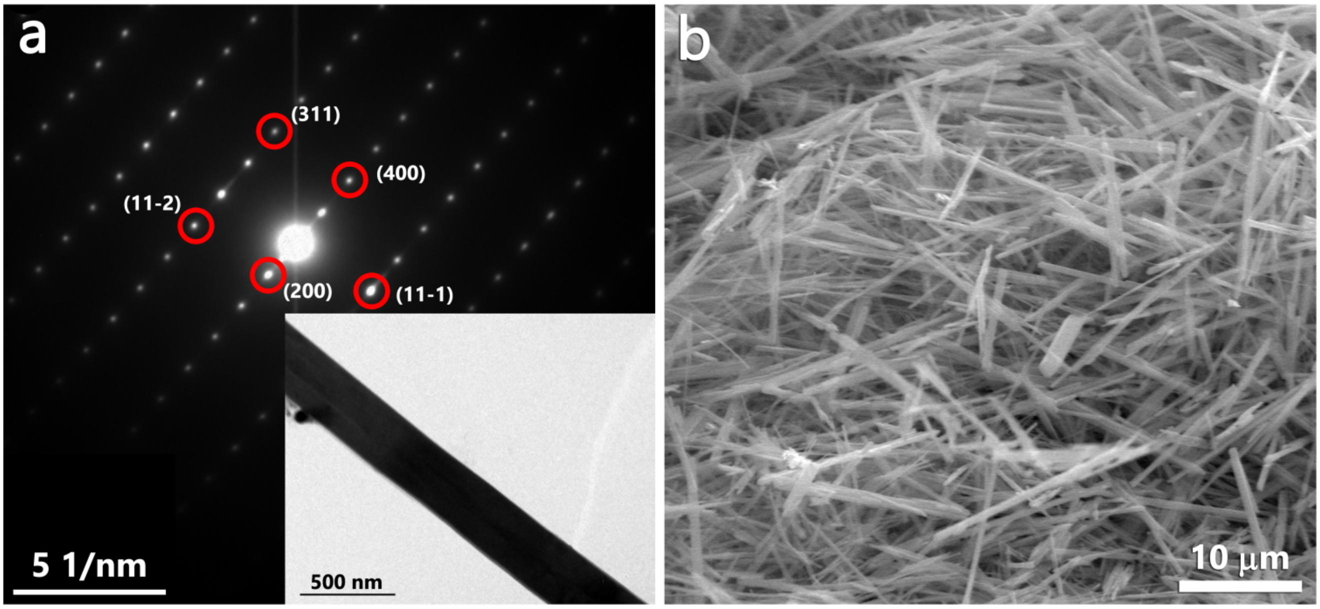

Figure S4. (a) Selected area electron diffraction of the nanowire in the inset. The single crystalline nature of the nanowire is illustrated. Reflections are indexed to the refined β'-$Li_xV_2O_5$ crystal structure from the high-resolution synchrotron powder diffraction data. (b) A SEM image of the nanowires upon re-incorporating Li-ions into the tunnel structure showing that the nanowire morphology is retained.

This journal is © The Royal Society of Chemistry 2012

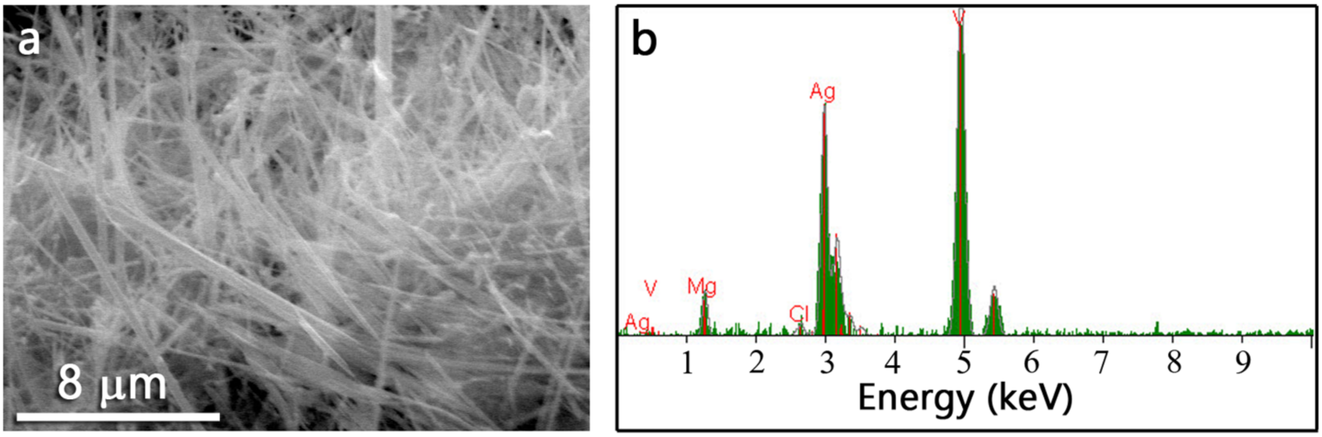

Figure S5. (a) SEM image of the β-$Mg_xV_2O_5$ nanowires illustrating retention of the nanowire morphology upon reaction of ζ-V_2O_5 with Mg nanoparticles. (b) EDX spectrum corresponding to the image in (a) showing incorporation of Mg into the tunnel structure. The residual silver is a result of AgCl that is also observed in the XRD patterns after the initial removal of Ag-ions from the β-$Ag_xV_2O_5$ starting compound.

This journal is © The Royal Society of Chemistry 2012
